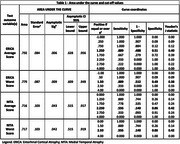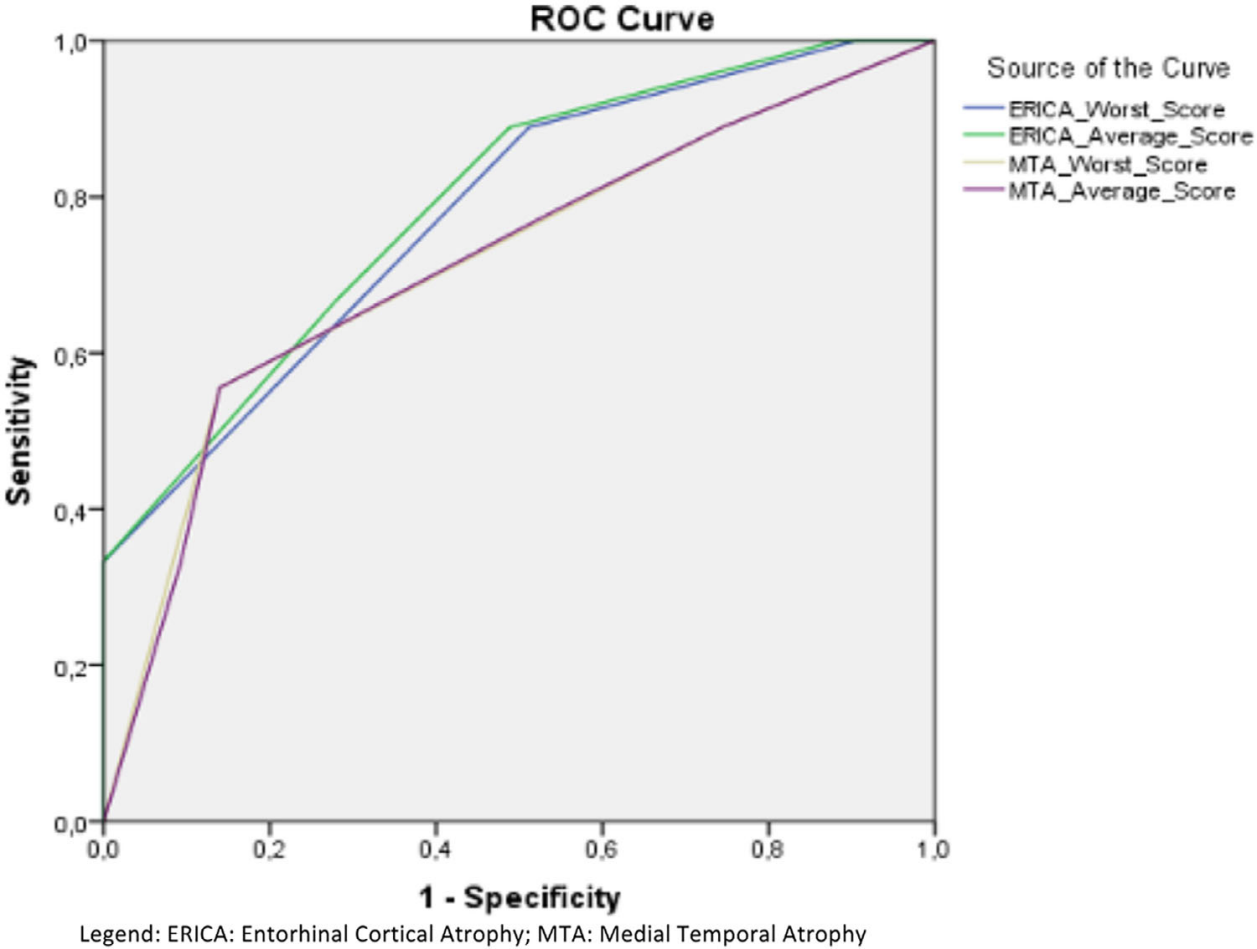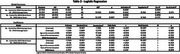# Accuracy of Visual Atrophy Scales on MRI to predict Mild Dementia in the Cog‐Aging Cohort Study

**DOI:** 10.1002/alz70856_107397

**Published:** 2026-01-09

**Authors:** Giovanna Correia Pereira Moro, Thaise Vallesca Queiroz, Gabriela Tomé Oliveira Engelmann, Carolina Andrade Koehne, Joice Coutinho de Alvarenga, Leticia Xavier Carneiro Calixto, Aline Siqueira de Souza, João Henrique Fonseca, João Marcos Silva Borges, Erika de Oliveira Hansen, Marco Aurelio Romano‐Silva, Maria Aparecida Camargos Bicalho, Jonas Jardim de Paula, Bernardo de Mattos Viana

**Affiliations:** ^1^ Cog‐Aging Research Group, Universidade Federal de Minas Gerais (UFMG), Belo Horizonte, Minas Gerais, Brazil; ^2^ Undergraduate Medicine, Faculty of Medicine, Universidade Federal de Minas Gerais (UFMG), Belo Horizonte, Minas Gerais, Brazil; ^3^ Older Adult's Psychiatry and Psychology Extension Program (PROEPSI), School of Medicine, Universidade Federal de Minas Gerais (UFMG), Belo Horizonte, Minas Gerais, Brazil; ^4^ Molecular Medicine Postgraduate Program, School of Medicine, Universidade Federal de Minas Gerais (UFMG), Belo Horizonte, Minas Gerais, Brazil; ^5^ Sciences Applied to Adult Health Postgraduate Program, School of Medicine, Universidade Federal de Minas Gerais (UFMG), Belo Horizonte, Minas Gerais, Brazil; ^6^ Geriatrics and Gerontology Center Clinical Hospital of Universidade Federal de Minas Gerais, Belo Horizonte, Minas Gerais, Brazil; ^7^ Neurotec R National Institute of Science and Technology (INCT‐Neurotec R), Faculty of Medicine, Universidade Federal de Minas Gerais (UFMG), Belo Horizonte, Minas Gerais, Brazil; ^8^ Department of Clinical Medicine, Faculty of Medicine, Universidade Federal de Minas Gerais (UFMG), Belo Horizonte, Minas Gerais, Brazil; ^9^ Department of Psychiatry, School of Medicine, Federal University of Minas Gerais, Belo Horizonte, Minas Gerais, Brazil

## Abstract

**Background:**

The Clinical Dementia Rating (CDR) is the most used categorization system for Alzheimer's Disease Dementia (ADD). In clinical practice, Magnetic Resonance Imaging (MRI) remains one of the most used methods for differential diagnosis and to improve accuracy, particularly with Visual Atrophy Scales (VAS).

**Objective:**

To conduct an accuracy analysis of two VAS to predict dementia with CDR1 in an Outpatient Memory Clinic.

**Methods:**

This is a cross‐sectional study, using data from the Cog‐Aging cohort study from 2019 to 2024. Participants underwent a comprehensive clinical, neuropsychological and neuroimaging assessment to determine CDR scores. Fifty‐two participants had MRI and assessments within 6 months. Participants were divided in Dementia (9) and Non‐Dementia (ND=43) groups. MRIs were assessed by two independent physicians, using The Entorhinal Cortical Atrophy (ERICA) scale and the Medial Temporal Lobe Atrophy (MTA) scale. They scored left and right, leading to an average score and a worst score. CDR Group scores were assessed with Mann‐Whitney. Furtheron, a Receiver Operating Characteristic (ROC) curve was done and cut‐off values were defined by Youden's J statistic. Finally, logistic regression models were done to predict CDR, considering VAS score, age and years of schooling. This study was approved by UFMG Ethics Committee.

**Results:**

Participants had a mean 76.28 years of age (SD 7.09) and median of 4.5 years of schooling (IQR 7.42). All VAS scores were significantly different between Dementia and ND. All scores had an Area Under the Curve (AUC) >0.715. ERICA average score had the highest AUC 0.792, achieving 88.9% sensitivity and 51% specificity at a cut‐off point of 1.5 and over. Finally, all scores were assessed by logistic regression adjusting for age and years of schooling, and only ERICA score's were significant (table 2). ERICA average score was the best model (Nagelkerke R^2^ 0.356; *p* =  0.006) with an Odds‐Ratio of 17.43.

**Conclusion:**

These findings suggest that ERICA average score was the most accurate method to detect Dementia in this sample, and also represented the best logistic regression model to predict Dementia when adjusted for years of schooling and age.